# miR-1226 detection in GCF as potential biomarker of chronic periodontitis: A pilot study

**DOI:** 10.4317/medoral.22329

**Published:** 2018-04-24

**Authors:** Pablo Micó-Martínez, José-Luis García-Giménez, Marta Seco-Cervera, Andrés López-Roldán, Pedro J. Almiñana-Pastor, Francisco Alpiste-Illueca, Federico V. Pallardó

**Affiliations:** 1 Dept. of Stomatology. Faculty of Medicine and Dentistry. University of Valencia. St/ Gascó Oliag, 1, 46010. Valencia, Spain; 2 Center for Biomedical Network Research on Rare Diseases (CIBERER). CIBER-ISCIII. St/ Álvaro de Bazán, 10, 46010. Valencia, Spain; 3 Dept. of Physiology. Faculty of Medicine and Dentistry. University of Valencia. Av/ Blasco Ibañez, 15, 46010. Valencia, Spain; 4 INCLIVA Health Research Institute. Av/ de Menendez y Pelayo, 4, 46010. Valencia, Spain; 5 Epigenetics Research Platform. CIBERER-UV. Av/ Blasco Ibañez, 15, 46010. Valencia, Spain

## Abstract

**Background:**

The study and identification of new biomarkers for periodontal disease, such as microRNAs (miRNAs), may give us more information about the location and severity of the disease and will serve as a basis for treatment planning and disease-monitoring. miRNAs are a group of small RNAs which are involved in gene regulation by binding to their messenger RNA target (mRNA). In this pilot study, the procedure for purifying miRNAs from gingival crevicular fluid (GCF) was, for the first time, described. In addition, the concentration of miRNAs in GCF was analyzed and compared between patients with moderate or severe chronic periodontitis (CP) and healthy controls.

**Material and Methods:**

GCF samples were collected from single-rooted teeth of patients with moderate or severe CP (n=9) and of healthy individuals (n=9). miRNAs were isolated from GCF using miRNeasy Serum/Plasma kit (Qiagen, CA. USA). Reverse transcription polymerase chain reaction (qRT-PCR) was used to determine the expression of a series of miRNAs candidates that are related to bone metabolism. The significance of differences in miRNA levels between both groups was determined using Mann-Whitney U test.

**Results:**

The results from this pilot study indicate that miRNAs can be isolated from GCF. Six different miRNAs were analyzed (miR-671, miR-122, miR-1306, miR-27a, miR-223, miR-1226), but only miR-1226 showed statically significant differences between the CP group and healthy controls (*p*<0.05). This miRNA was downregulated in patients with CP.

**Conclusions:**

Within the limitations of the present study, it may be concluded that miR-1226 can be a promising biomarker for periodontal disease, adding relevant information to common clinical parameters used for diagnosis and prognosis of periodontitis.

** Key words:**Small interfering RNA, biomarkers, periodontal diseases, reverse transcriptase polymerase chain reaction.

## Introduction

Predominantly Gram-negative anaerobic or facultative bacteria within the subgingival biofilm are the primary etiological agents of periodontal diseases, nevertheless, the majority of periodontal tissue destruction is caused by an inappropriate host response to those microorganisms and their products (1).

GCF is a transudate that is released into the gingival sulcus of the teeth. It is mainly composed of polymorphonuclear leukocytes (PMNs), serum proteins, bacteria, tissue breakdown products, enzymes, antibodies and numerous inflammatory mediators and nucleic acids. GCF collection is a non-invasive and simple procedure and is especially useful for identifying people at risk for initiation or progression of periodontitis and for monitoring the response to periodontal therapy (2).

Epigenetic regulation can exert a narrow control over the expression of key genes which underlies the pathophysiology of periodontal disease (i.e. inflammation, bone resorption, etc.) (3). There is evidence suggesting that chronic inflammation and bacterial infection alters DNA methylation of genes codifying for pro-inflammatory (i.e.IL-1, IL-6, IFN-γ, TNF-α) and anti-inflammatory cytokines (i.e., IL4, IL-10, IL-11, etc.) (4). Furthermore, Zhang *et al.* observed a hypomethylation of the inflammatory gene IFN-γ (5) and also a hypermethylation of TNF-α (6). In the same study, they observed in inflamed gingival tissues from patients with CP, a decreased expression of COX-2 due to a hypermethylation of its promoter, results that were also demonstrated by Loo *et al.* (7). Besides DNA methylation, histone post-translational modifications (HPTMs) also regulate epigenetic mechanisms. In fact, histone deacetylase inhibitors (HDACi), such as 1179.4b and MS-275, have shown promising therapeutic properties against bone loss produced on Porphyromonas gingivalis-inoculated mice (8), suggesting that HPTMs regulate the expression of key genes involved in periodontal disease.

Besides the above-mentioned epigenetic mechanisms, non-coding RNAs (i.e. long non-coding RNAs and miRNAs) also participate in the epigenetic control of gene expression. However, as far as we know, epigenetic regulation by miRNAs has not yet been studied in periodontal disease using GCF.

miRNAs are a new and promising potential biomarker for diagnosis and prognosis of many diseases (9) because they act as signaling molecules and participate in many biological processes, such as cellular development, differentiation, and apoptosis. The high stability of circulating miRNAs in a RNase-rich environment such as the bloodstream (10), make these biomolecules an optimal source for the identification of candidate biomarkers. In fact, miRNAs have demonstrated their value as biomarkers in a wide variety of human diseases (9).

miRNAs are a large family of short non-coding RNAs (17-25 nucleotides) which are involved in gene regulation by binding to their messenger RNA target (mRNA). In the periodontum, miRNAs may play key roles in periodontal tissue development and homeostasis and during the loss of periodontal tissue integrity as a result of periodontal disease (11). In addition, they have been proposed as important contributors to bone morphogenesis and osteoclastogenesis (OsteomiRs) (12), making them interesting biomolecules for the study of molecular causes of periodontal diseases.

These findings prompted us to investigate miRNA extraction and quantification from an easily collected transudate, the GCF. In addition, the concentration of miRNAs that have been related to bone metabolism was analyzed and compared between patients with moderate or severe chronic periodontitis and healthy controls.

## Material and Methods

-Patient and site selection

Nine healthy individuals (four males and five females, aged 25 to 60 years) and nine patients with moderate or severe CP (three males and six females, aged 36 to 61 years) who were referred to the Department of Periodontology at the Faculty of Medicine and Dentistry, University of Valencia, Valencia, Spain, participated in this pilot study. The study was conducted from January to July 2016. Every patient and healthy individual enrolled in the investigation provided written informed consent. In addition, the research related to human use has been complied with all the relevant national regulations, institutional policies and in accordance the tenets of the Helsinki Declaration, and has been approved by the Experimental Research Ethics Committee of the University of Valencia.

For each individual, pocket depth (PD), recession, clinical attachment level (CAL) and bleeding on probing (BP) values were measured with a periodontal probe. A single calibrated examiner assessed these clinical parameters. Healthy individuals (control group) did not present any sign or symptom compatible with periodontal disease (PD<3mm, CAL<3mm and no radiographic evidence of alveolar bone breakdown). On the other hand, the test group consisted of patients with moderate or severe CP (based on the Classification of Periodontal Diseases and Conditions of Armitage, 1999) (13) with at least one single-rooted tooth with CAL≥6mm and probing depth ≥5mm. Exclusion criteria included: 1) smoking; 2) patients diagnosed with chronic periodontitis but without one single-rooted tooth with CAL≥6mm and probing depth ≥5mm.; 3) patients diagnosed with aggressive periodontitis; 4) presence of any systemic disease or use of drugs that affect bone metabolism (osteoporosis, arthritis, hormonal therapy, bisphosphonates, anti-inflammatories, immunosuppressants) 5) use of antibiotics, anti-inflammatories or contraceptives within the last three months; 6) primary or secondary occlusal trauma in teeth included for the study; 7) patients who had received periodontal treatment within the last 6 months; 8) patients undergoing orthodontic treatment; 9) pregnant or lactating women; 10) patients with a periapical or periodontal abscess around the tooth used for the study.

In both groups, a GCF sample was taken from a single-rooted tooth (in test group, from teeth with CAL≥6mm), due to its easy access and to avoid errors associated to GCF sample collection.

-GCF collection

Before placing the absorbing paper strip within the sulcus, supragingival plaque was removed, and relative isolation was performed using cotton rolls and aspiration to prevent saliva contamination. Subsequently, the filter paper strip Periopaper® (Oraflow, New York, NY, USA) was placed in the gingival sulcus until resistance was felt and left in this position for 30 seconds (14). If the paper was bloodstained or contaminated with saliva or detritus, the sample was excluded and the procedure was repeated. Finally, the filter paper strip was introduced in a sterile Eppendorf tube and stored at -80ºC.

-RNA extraction and quantification from GCF 

Periopapers® were incubated in Phosphate-saline buffer (PBF) solution pH 7 for 30 min at room temperature. In order to recover all the solution contained in the strip, each sample was centrifuged at 500 rpm for 10 minutes and stored again at -80ºC until RNA extraction. Cell-free total RNA (including miRNAs) was isolated from 200 uL of PBS solution containing the biological material from the Periopaper® using miRNeasy Serum/Plasma kit (Qiagen, CA. USA). RNA was eluted with 25 µL of RNAse-free water. Cell-free total RNA (including miRNAs) concentration was quantified using NanoDrop ND 2000 UV-spectrophotometer (Thermo Scientific, Wilmington, DE, USA) and the sample quality was measured with the Small RNA Assay, Agilent 2100 Bioanalyzer (Agilent Technologies).

-miRNAs signature prediction for CP diagnosis

In order to identify which miRNAs would be interesting to study, a literature review was conducted using Pubmed database, on the relevant biological pathways related to bone metabolism and periodontitis. Subsequently, from the selected manuscripts, key pathways represented in KEGG (Kyoto Encyclopedia of Genes and Genomes) were selected. Then, mirPath v3 KEGG Reverse Search (selecting the method TarBase v7.0) was used to identify miRNAs that target key genes of those pathways (15).

Furthermore, we also included blood-circulating miRNAs which had been related to bone metabolism regulation, based on a previous study carried out on patients with idiopathic scoliosis (16).

We finally chose those miRNAs which were found at least 5 times in each KEGG pathway and that coincided with García-Giménez’s study (16). The selected miRNAs are shown in  Table 1.

**Table 1:**
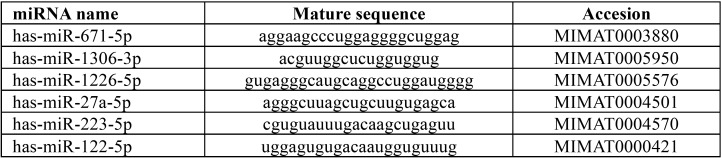
Selected miRNAs as biomarkers for periodontal disease.

-Quantitation of miRNAs by RT-PCR 

Reverse transcription (RT) reactions were performed using TaqMan miRNA Reverse Transcription kit, miRNA-specific stem-loop primers (Part No. 4366597, Applied Biosystems, Inc) and 100 ng of input cell-free RNA in 15 µL RT reaction. Real-time PCR reactions were performed in triplicate, in scaled-down 10 µL reaction volumes using 5 µL TaqMan 2x Universal PCR Master Mix with No UNG, 0.5 µL TaqMan Small RNA assay (20x), hsa-miR-27a-5p (002445), hsa-miR-223-5p (002098), hsa-miR-671-5p (197646_mat), has-miR-122-5p (002245), hsa-miR-1226-5p (002758), hsa-miR-1306-3p (241056_mat) and hsa-miR-16-5p (000391) as endogenous control gene ( Table 1), 3.5 µL of nuclease free water and 1 µL of RT product. Real-time PCR was carried out on Applied BioSystems 7900HT thermocycler (Applied Biosystems. Inc, CA; USA) programmed as follows: 50ºC for 2 minutes, 95 ºC for 10 minutes followed by 45 cycles of 95ºC for 15 seconds and 60ºC for 1 minute. One of the most stable counting reads miRNAs and previously used as endogenous control (17), miRNA hsa-miR-16-5p (000391), was used to normalize the expression data using the delta-delta CT method (2-ΔΔCT).

-miRNA target prediction and over-representation analysis

There are a large number of potential mRNA target sites for any given miRNA. The computational approach to predict miRNAs targets facilitates the selection process of specific targets. For our study, DIANA-microT web server v5.0 (18) was used, suited to identify those genes whose expression appeared to be controlled by the statistically significant miRNAs. For this purpose we used the following default parameters: DIANA TarBase v.7.0, with a *p*-value threshold of 0.001, and microT with a threshold of 0.8. To reduce false positive miRNA targets, we applied a false discovery rate (FDR) correction. The algorithm utilized in this analysis consisted on one-tailed Fisher’s exact test.

-Statistical analysis

The mean was taken as the measurement of the main tendency, and the standard deviation was taken as the dispersion measurement for the statistical analysis of results. Mann-Whitney U analysis was performed to compare both groups of patients and controls. A *p*-value of <0.05 was considered statistically significant.

## Results

There were 6 (66.7%) females and 3 (33.3%) males in the test group, and 5 (55.6%) females and 4 (44.4%) males in the healthy control group. The mean age for the chronic periodontitis group was 50.44±8.09 (36-61) years old and 33.33±12.05 (25-60) years old for the healthy group. The mean values of the clinical attachment level of the single-rooted teeth were 7.00±1mm for the test group and 2.00±0.71mm for the control group. These demographic and clinical parameters are summarized in  Table 2.

**Table 2:**
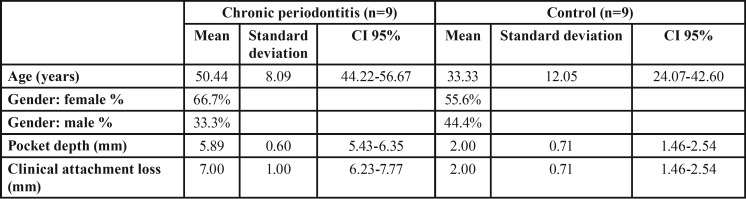
Demographic and clinical characteristics of test and control group.

-miRNAs can be isolated from GCF

From each sample, 10 ng of total RNA was analyzed using the Agilent Small RNA Assay. This assay shows the distribution of small RNA fragments (including miRNAs) ranging in size from 6 to 150 nt, thus allowing the detection of different RNA families.

**Figure 1:**
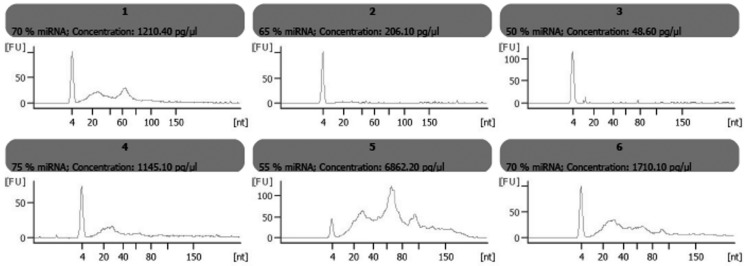
Electropherograms of smallRNAs isolated from GCF of 3 randomized patients and 3 randomized controls. Elecropherograms showed differences among them in smallRNA distribution and miRNA enrichment percentage.

This investigation shows that smallRNAs can be isolated from GCF. The electrophoretic profile of RNA from GCF showed different bands probably due to the presence of different RNA families: miRNA molecules (10 to 40 nt, mainly concentrated at 20 nt), small RNA fragments (between 6 to 150 nt), snoRNA fragments (60-150 nt) and pre-miRNAs (90-150 nt). Different patterns in elecropherograms from 6 randomized samples obtained from patients (samples 1, 2 and 3) and from controls (samples 4, 5, and 6) were observed, indicating differences in smallRNA distribution and miRNA enrichment percentage in the whole recovered sample (Fig. 1).

-Selected miRNAs for the study

The following six miRNAs were found at least 5 times in each KEGG pathway and coincided with García-Giménez’s study(16), and thus were selected for the study: miR-671, miR-122, miR-1306, miR-223, miR-27a and miR-1226.

-miR-1226-5p identifies patients with periodontal disease

Reverse transcription polymerase chain reaction (RT-PCR) was used to determine the relative expression levels of the six representative miRNAs selected for the study ( Table 3).

**Table 3:**
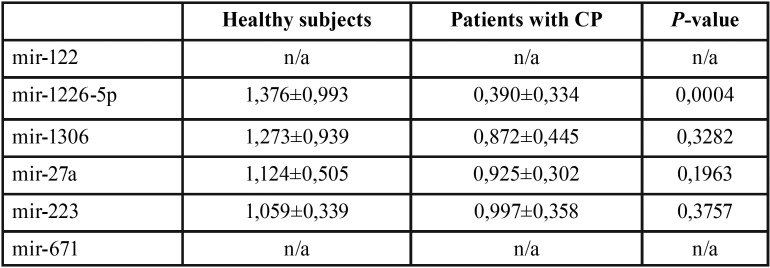
Relative quantification of miRNAs by real-time RT-PCR. Mean and standard deviation (mean±sd) of miRNAs relative expression (2-ΔΔCT) in healthy subjects and patients with CP. *P*-value obtained comparing miRNAs relative expression of healthy subjects and patients with CP using U of Mann-Whitney analysis. n/a: not data.

**Figure 2:**
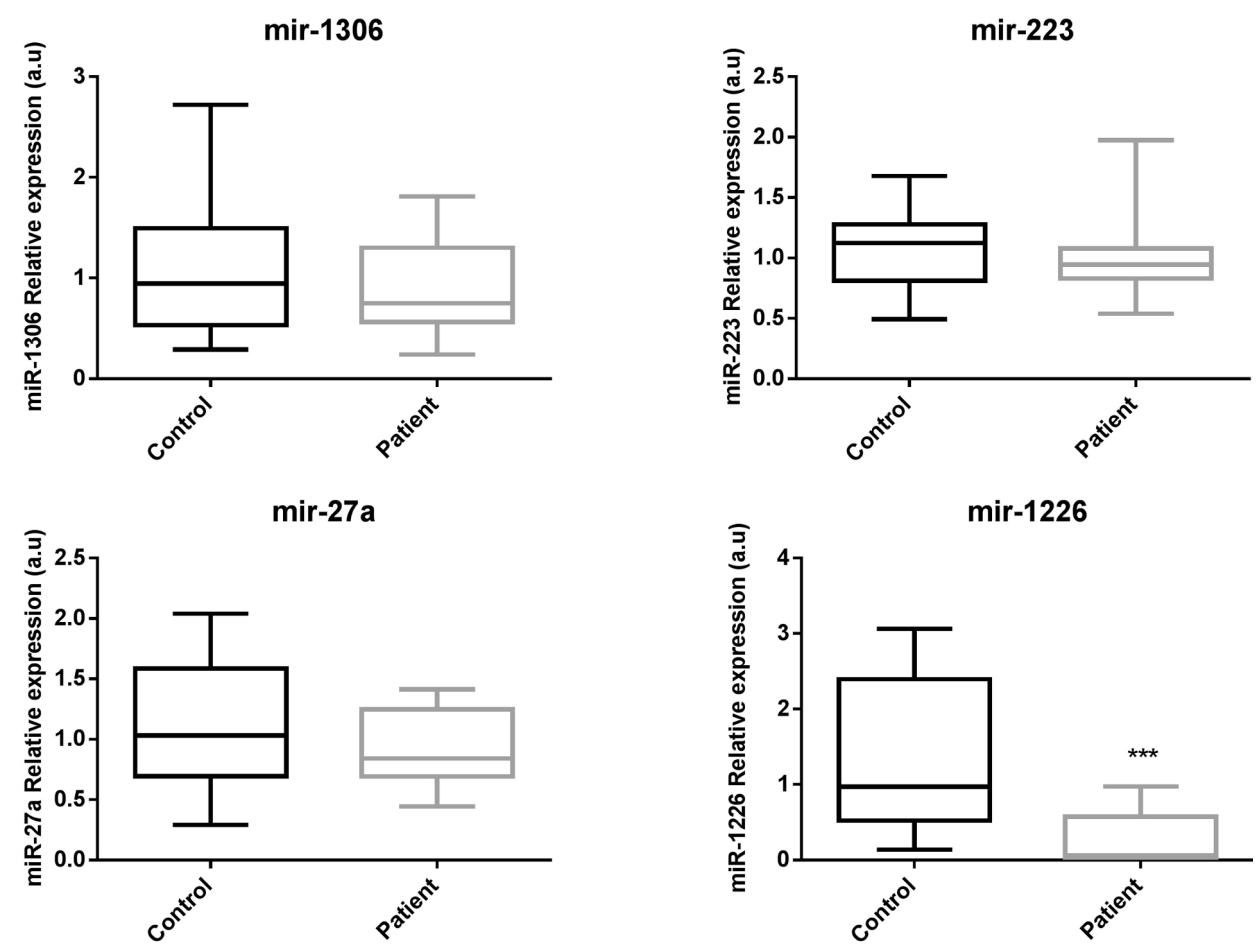
Relative expression levels of miRNAs detected in GCF of patients with CP compared to healthy subjects. Black bars correspond to control group and grey bars to test group. U of Mann-Whitney analysis was applied. *p*< 0.05 was considered to be statistically significant. miR-1226 was significantly downregulated in test group compared to controls.

miR-671 and miR-122 were not detected in GFC, unlike the other 4 miRNAs studied. In addition, differences in expression levels of miR-1306 and miR-223 between patients and controls were not found (Fig. 2 and  Table 3). On the other hand, regarding to miR-27a, a tendency to be downregulated in patients with CP was observed, nevertheless these differences were not statistically significant. However, miR-1226-5p was found to be 15.8-fold downregulated (*p*<0.05) in patients with periodontal disease suggesting that this miRNA could be a feasible biomarker of the disease (Fig. 2).

-miRNA targets analysis 

miR-1226-5p was the only epigenetic biomarker that showed statistically significant differences between healthy subjects compared to patients in regards to relative expression levels. Therefore, we only searched for target genes whose expression was controlled by this miRNA. In this regard, the proteins calreticulin and mucin 1 were identified as target genes of this miRNA, by DIANA-microT web server v5.0.

## Discussion

The identification of new epigenetic biomarkers may clearly contribute to improve clinical diagnosis and prognosis of many human diseases, such as periodontitis.

miRNAs have been considered promising biomarkers (9) due to its high stability in different biofluids commonly used in analytical assays (9,10).

To our knowledge, this is the first work that have reported the possibility of detecting and measuring by qRT-PCR techniques, miRNAs in GCF, in order to identify periodontitis biomarkers. Our first result indicated that smallRNAs can be isolated from GCF using similar procedures to those used for other biological fluids such as plasma. Because different elecropherogram patterns were found in the same group (patients or controls), we suggest that differences in smallRNA profile are not due to the pathological process itself, but to the differences in small RNA biological profiles of each individual, changes in the ability of Periopaper® to absorb smallRNA species, and/or different recovery efficiencies of smallRNA from Periopaper® during smallRNA purification procedure.

In order to identify miRNAs associated to periodontal disease, we measured 6 miRNAs related to bone metabolism, some of them (i.e. miR-27a and miR-223) also known as osteomiRs (12). In our study, miR-1226 was the only miRNA which was significantly downregulated in patients with CP.

In addition, we used in silico studies (DIANA-microT web server v5.0) to identify miR-1226 targets such as CALR and MUC1. Particularly, this miRNA targets the 3’ UTR of CALR messenger so its function will be blocked (19). CALR is a multifunctional protein whose main functions are Ca2+ storage and gene expression regulation when is located in the nucleus. CALR expression can affect retinoic acid-dependent differentiation and inhibit vitamin D-induced stimulation of osteocalcin expression, so it can inhibit mineralization (19). Therefore, downregulation of miR-1226 may produce CALR overexpression contributing to bone mineralization decrease.

On the other hand, MUC1 acts as a tumor suppressor as it prevents aberrant upregulation of genes related to cell acquisition of malignant phenotype (20). MUC1 overexpression gives rise to two protein subunits that can interact with members of the receptor tyrosine kinase family and induce cell transformation (21). On the other hand, MUC1 can also control Wnt/β-catenin pathway. MUC1-C72 amino acid cytoplasmic domain (MUC1-CD) interacts with different effectors, promoting cell survival and proliferation, apoptosis and inflammation in different cells, including osteoblasts. For example, MUC1 blocks GSK3B phosphorylating β-catenin, therefore avoids β-catenin degradation (21). This is very important because Wnt signaling plays an essential role in bone metabolism by controlling the stem cells differentiation into mature osteoblasts or osteoclasts (22,23).

MUC1 can also be involved protein p53 transcription regulation after genotoxic stress, inhibiting p53-mediated apoptosis and promoting cell cycle arrest (24), therefore MUC1 can block cell cycle and proliferation in osteoblasts cells.

In addition, MUC1 also activates IκB (Kinase beta complex) (25) therefore releasing IκBα activating NF-κB p65/RelA (26), so contributes to the activation of canonical NF-κB pathway (27). This NF-κB regulation could be of special interest not only for its role in osteoclast formation in presence of receptor activator of NF-κB ligand (RANKL) and TNF, but also because it has been recently described the essential dual role of NF-κB activating or inhibiting cytokine-mediated osteoclast formation by regulating the key factor NFATc1 expression (27).

Furthermore, we reviewed the literature in order to identify other possible roles or mechanisms in which miR-1226 participates in regulating bone metabolism. Interestingly, we found out that miR-1226 was one of the miRNAs able to differentiate acute from chronic disease cases of Brucellosis, being this miRNA specifically downregulated in chronic cases (28). In brucellosis disease, a serious loss of bone occurs (28). One of the reasons is the damage that Brucella produces on osteoblast function. Furthermore, Brucella infection induces an increase in the number of osteoclasts, reduces osteoblast differentiation and contributes to its apoptosis (29), therefore resulting in bone resorption.

In the present study, we described the procedure for purifying miRNAs from GCF. In addition, this pilot study shows that miR-1126 can provide clinicians another tool to identify susceptible patients and may add relevant information to common clinical parameters used for diagnosis and prognosis of periodontitis. These preliminary results encourage further investigation with a larger sample size to determine the effectiveness of miR-1226 as a valuable biomarker for periodontitis.
